# Challenges in Nanofiber Formation from NADES-Based Anthocyanin Extracts: A Physicochemical Perspective

**DOI:** 10.3390/ma18194502

**Published:** 2025-09-27

**Authors:** Paulina Wróbel, Katarzyna Latacz, Jacek Chęcmanowski, Anna Witek-Krowiak

**Affiliations:** 1 Department of Engineering and Technology of Chemical Processes, Faculty of Chemistry, Wroclaw University of Science and Technology, Gdanska 7/9, 50-344 Wroclaw, Poland; 2 Faculty of Chemistry, Wroclaw University of Science and Technology, 50-370 Wroclaw, Poland; 272043@student.pwr.edu.pl; 3 Department of Advanced Material Technologies, Faculty of Chemistry, Wroclaw University of Science and Technology, Smoluchowskiego 25, 50-370 Wroclaw, Poland; jacek.checmanowski@pwr.edu.pl

**Keywords:** electrospinning, natural deep eutectic solvents, anthocyanins, sodium alginate, poly(ethylene oxide), freshness indicator

## Abstract

This study explores the challenge of using anthocyanin-rich natural deep eutectic solvent (NADES) extracts to produce electrospun nanofibers for biodegradable freshness indicators. Red cabbage was extracted with two choline chloride-based NADESs (with citric or lactic acid), modified with 10–50% ethanol to lower viscosity, and compared with a standard 50% ethanol-water solvent. The citric acid NADES with 30% ethanol gave the highest anthocyanin yield (approx. 0.312 mg/mL, more than 20 times higher than the ethanol extract at approx. 0.014 mg/mL). For fiber fabrication, a polymer carrier blend of poly(ethylene oxide) (PEO) and sodium alginate (Alg) was employed, known to form hydrogen-bonded networks that promote chain entanglement and facilitate electrospinning. Despite this, the NADES extracts could not be electrospun into nanofibers, while the ethanol extract produced continuous, smooth fibers with diameters of approximately 100 nm. This highlights a clear trade-off; NADESs improve anthocyanin recovery, but their high viscosity and low volatility prevent fiber formation under standard electrospinning conditions. To leverage the benefits of NADES extracts, future work could focus on hybrid systems, such as multilayer films, core-shell fibers, or microcapsules, where the extracts are stabilized without relying solely on direct electrospinning. In storage tests, ethanol-extract nanofibers acted as effective pH-responsive indicators, showing visible color change from day 4 of meat storage. At the same time, alginate films with NADES extract remained unchanged after 12 days. These results highlight the importance of striking a balance between chemical stability and sensing sensitivity when designing anthocyanin-based smart packaging.

## 1. Introduction

Anthocyanins are a category of polyphenolic metabolites belonging to the flavonoid family, primarily found in glycosylated forms. Their fundamental chemical structure originates from the flavylium ion (2-phenylbenzopyrylium), which is commonly modified by hydroxyl or methoxyl substituents and covalently attached to sugar residues like glucose and rhamnose [1]. These structural features contribute to their chromophoric properties and determine their stability, solubility, and reactivity. Anthocyanins are major contributors to the coloration of plant organs, producing hues that range from red through purple to blue. The observed color is susceptible to physicochemical parameters, including pH, polarity of the surrounding medium, metal ion complexation, and co-pigmentation [2,3]. In aqueous solutions, anthocyanins undergo reversible structural transformations that are strongly pH-dependent. At acidic pH, the predominant species is the red-colored flavylium cation, while increasing the pH leads to the formation of quinoidal bases, which exhibit blue to purple hues. Under near-neutral or alkaline conditions, the molecules may further convert into colorless chalcone forms. The ability of anthocyanins to reversibly transition between distinct molecular forms through protonation–deprotonation mechanisms underlies their functionality as environmentally responsive chromophores in pH-sensitive systems [2]. These compounds have traditionally been used as colorants; however, due to their biological properties, including anti-inflammatory, antimicrobial, and antioxidant activities, they also show great potential for applications in medicine, packaging, and the food industry [4]. Anthocyanins, owing to their color variations based on pH, are exemplary candidates for application as freshness indicators in intelligent food packaging. Their capacity to react to biochemical alterations linked to spoiling, including pH variations or the detection of volatile amines, facilitates real-time assessment of product quality [5]. Anthocyanins exhibit sensitivity to light, heat, and oxidation, hence constraining their stability in practical applications. Stabilization techniques to enhance performance include pH control, acylation, co-pigmentation, and encapsulation [1,6,7,8,9]. Electrospinning enables efficient incorporation of anthocyanins into nanofibers, enhancing their protection, controlled release, and functionality as colorimetric indicators [10]. 

The efficient extraction of anthocyanins is essential for the development of functional materials, as it directly influences their purity, stability, and bioactivity. Traditional solvents like ethanol and methanol are frequently employed; nonetheless, their drawbacks regarding selectivity, volatility, and sustainability have prompted increased exploration of alternative, environmentally friendly extraction techniques. Natural deep eutectic solvents (NADES), especially those formulated with choline chloride (ChCl) and organic acids or polyols, demonstrate significant efficacy in the extraction and stabilization of anthocyanins. These solvents result from distinct hydrogen bonding interactions between a hydrogen bond donor (HBD) and a hydrogen bond acceptor (HBA), producing a eutectic mixture with a melting point considerably lower than that of the individual components. NADESs, characterized by tunable polarity, low vapor pressure, and inherent biodegradability, serve as a sustainable and selective medium for the extraction of polar phytochemicals. NADESs, characterized by tunable polarity, low vapor pressure, and biodegradability, represent a sustainable and selective medium for extracting polar phytochemicals [11]. 

Moreover, their somewhat acidic nature creates a conducive environment that preserves the structural integrity of anthocyanins during extraction [12]. NADES not only improves extraction efficiency but also boosts the stability of the recovered anthocyanins [13,14,15]. ChCl-based NADESs, especially those using citric or lactic acid, are among the most efficient and extensively researched methods for anthocyanin extraction, providing high yields, enhanced pigment stability, and compatibility for scaling up. Their recorded efficiency across several plant matrices robustly endorses their choice for comparative assessment in this work [11,16,17,18]. Notwithstanding the growing interest in NADESs, obstacles persist in their actual implementation. Numerous NADESs demonstrate elevated viscosity, potentially obstructing mass transfer and constraining extraction efficiency [12]. Furthermore, their hygroscopic properties and low volatility may present difficulties in downstream processing applications, especially in electrospinning, which often necessitates low-viscosity, rapidly evaporating solvent systems.

In this work, we employed the electrospinning technique, which uses a strong electric field to produce ultrathin fibers from a polymer solution. Electrospinning was selected for its ability to incorporate sensitive compounds such as anthocyanins into stable nanofibrous mats with high surface area and porosity [19]. In this case, the goal is not controlled release, but rather a rapid and visible color change, which is facilitated by the thin fibers and their porous structure, making them well-suited for freshness indicator applications. Considering the established efficacy of NADESs in enhancing anthocyanin extraction and stabilization, this study seeks to examine the compatibility of these extracts with electrospinning. This method has demonstrated efficacy in the fabrication of pH-sensitive indicator fibers utilizing conventional solvents. Currently, only one study has documented the successful integration of NADES-extracted anthocyanins into electrospun fibers. In that work, Vannuchi et al. (2024) extracted anthocyanins from jussara pulp using a (chlorine-chloride: xylitol, 5:2) NADES system and incorporated the extract into polyethylene oxide (PEO)-based electrospun fibers [20]. The resultant nanofibers demonstrated improved encapsulation efficiency and augmented pigment stability in simulated gastrointestinal conditions. Notwithstanding these encouraging outcomes, the general applicability of this methodology remains ambiguous, as no research has yet used this technique on alternative anthocyanin-rich plant matrices or varied NADES compositions.

This study attempts to fill the research gap by comprehensively assessing the suitability of NADES-extracted anthocyanins for electrospinning applications, despite their superior extraction efficiency and stabilizing qualities. This study offers mechanistic insights into how the physicochemical features, including viscosity and hygroscopicity, of NADES-based systems may impede fiber creation, thereby defining critical limitations and informing future applications in intelligent food packaging. This study’s innovation is in assessing two distinct solvent systems for extraction efficiency and their possible compatibility with electrospinning, utilizing red cabbage as a model source of structurally varied anthocyanins [21]. This technique represents a new effort to combine green extraction technologies with nanofiber production, given the intrinsic plasticizing capabilities of NADESs that may facilitate fiber creation. Red cabbage, selected for its high anthocyanin content and pigment diversity, was extracted using ethanol-modified ChCl-based NADES (at 10–50% EtOH) and a standard 50% ethanol solution for comparative analysis of extraction performance and suitability for electrospinning. The experimental results showed that, although the extraction efficiency was superior, the viscosity and hygroscopicity of the systems containing NADES impeded the production of continuous and stable fibers, revealing notable physicochemical limitations of this method. However, achieving successful fiber formation requires precise control of solution properties such as viscosity, conductivity, and surface tension. The high water content of the spinning mixture often hinders it. To mitigate these limitations, we employed a biopolymer blend of sodium alginate and poly(ethylene oxide) as the carrier matrix for electrospinning. PEO promotes the formation of uniform fibers, while Alg enhances film-forming capacity and can be ionically crosslinked to stabilize the resulting structures. These polymers are known to establish compatible hydrogen-bonded networks [22], enabling cohesive spinning solutions even in aqueous systems. Furthermore, ethanol was introduced as a cosolvent in the NADES formulations to reduce both boiling point and viscosity, thereby enhancing the electrospinnability of the otherwise water-rich extracts.

This study seeks to (i) evaluate the efficiency of anthocyanin extraction from red cabbage utilizing two NADES formulations, specifically ChCl with citric acid and with lactic acid, modified with varying ethanol concentrations; and (ii) examine the applicability of the resultant extracts for electrospinning. Trials of electrospinning were performed to evaluate the capability of NADES-based solutions to produce continuous fibers. The findings are analyzed regarding extraction performance, solvent composition, physicochemical properties, and critical factors influencing electrospinnability. Additionally, selected materials incorporating ethanol- and NADES-based extracts were subjected to colorimetric testing under real meat storage conditions to preliminarily assess their potential as pH-sensitive freshness indicators, offering insight into the functional trade-offs between extraction efficiency, processability, and sensory responsiveness.

## 2. Materials and Methods

### 2.1. Materials

Red cabbage and chicken breast meat were purchased from a local shop (Wroclaw, Poland). Sodium alginate (Alg), polyethylene oxide (1,000,000 g/mol), choline chloride, gallic acid monohydrate, hydrochloric acid (fuming ≥ 37%), and sodium carbonate were purchased from Sigma Aldrich (St. Louis, MO, USA). Lactic acid, glycerol anhydrous and Folin-Ciocalteu reagent were from Chempur (Piekary Slaskie, Poland). Citric acid monohydrate, potassium chloride and ethanol (99.9%) were purchased from Stanlab (Lublin, Poland). Sodium acetate and calcium chloride anhydrous were from EUROCHEM BGD (Tarnow, Poland).

### 2.2. Solvent Selection and Preparation

Anthocyanins are very sensitive to factors such as light, heat, and oxygen; thus, acidic solvents were used to help the pigments stay stable during extraction. Two NADES formulations were assessed: first, consisting of ChCl, citric acid and water (1:2:6, mol/mol/mol), and second, comprising ChCl and lactic acid (1:1, mol/mol). Due to the elevated viscosity and hygroscopic nature of pure NADES, which may impede extraction efficiency and subsequent electrospinning, ethanol (99.99%) was integrated into the solvent systems at varying weight ratios (10%, 30%, and 50% *w*/*w*). The incorporation of ethanol sought to diminish viscosity, improve mass transfer, and elevate the electrospinnability of the resultant anthocyanin-rich solutions.

In total, six mixed solvent systems were prepared (Table 1): 0CA, 1CA, 2CA, and 3CA (ChCl:citric acid:water (1:2:6) with 0%, 10%, 30%, and 50% ethanol, respectively) and 1LA, 2LA, and 3LA (ChCl:lactic acid (1:1) with 10%, 30%, and 50% ethanol, respectively). For comparison, a reference extraction was also carried out using a conventional hydroalcoholic solvent consisting of 50% ethanol in water (designated Et).

### 2.3. Antocyanins Extraction

Dried and powdered red cabbage leaves were extracted with each solvent at a plant material to solvent ratio of 1:10 (*w*/*w*). The mixtures were agitated in a PSU-20i orbital shaker (Biosan, Riga, Latvia) for 30 min at 175 rpm at ambient temperature and subsequently subjected to centrifugation at 6000 rpm for 10 min. The supernatants were filtered and preserved at 4 °C for subsequent analysis.

### 2.4. Total Polyphenol Content (TPC) Determination

The total phenolic content (TPC) of each extract was determined using the Folin–Ciocalteu colorimetric method with slight modifications. Briefly, 0.1 mL of appropriately diluted extract was mixed with 7.9 mL of water and 0.5 mL of 10% (*v*/*v*) Folin–Ciocalteu reagent. After 5 min, 1.5 mL of saturated sodium carbonate solution was added. The mixture was incubated in the dark at room temperature for 30 min, and the absorbance was then measured at 765 nm using a S1020 UV-VIS spectrophotometer (Techcomp, Hong Kong, China). Gallic acid was used as the calibration standard, and the results were expressed as milligrams of gallic acid equivalents per milliliter of extract (mg GAE/mL). 

### 2.5. Total Anthocyanin Content Determination (TAC)

The total anthocyanin content (TAC) was determined using the pH differential spectrophotometric method as previously described [23]. Each extract was diluted with two buffer systems: a 0.025 M potassium chloride buffer (pH 1.0) and a 0.4 M sodium acetate buffer (pH 4.5). Absorbance readings were taken at 520 nm and 700 nm for both pH values using a UV-Vis spectrophotometer. Sample dilutions were adjusted to ensure that absorbance values fell within the range of 0.1 to 1.0. The TAC was calculated as cyanidin-3-glucoside equivalents (mg/mL).

### 2.6. Electrospinning Solutions Preparation

The solutions of PEO (2% *w*/*v*) and sodium alginate (Alg) (4% *w*/*v*) were prepared by dissolving them in distilled water at 80 °C and at room temperature, respectively. Then, each extract ((E)Et, (E)1CA, (E)2CA and (E)3CA) was added to the mixture of PEO/Alg (in a mass ratio of 3:1) to obtain a final extract content of 5% in the electrospinning solutions, and the mixtures were stirred at room temperature until complete homogenization. In the case of (E)Et, an additional concentration of 20% of the extract was also prepared.

### 2.7. The Rheological Properties of Electrospinning Solutions

The rheological properties of three solutions: (S)Et5%, (S)Et20% and (S)2CA5% were evaluated using a ViscoQC 100 rotational viscometer (Anton Paar, Graz, Austria) with a spindle type RH2, across a rotational speed range of 10–80 rpm.

### 2.8. Electrospinning Process

The electrospinning process was employed to produce nanofibers from PEO-Alg and anthocyanin extracts using a Professional Lab Device electrospinning machine (DOXA Microfluids, Malaga, Spain). The polymer solution was pumped through a syringe with a 20-gauge needle. All samples were electrospun under a fixed set of conditions (20 cm needle-to-collector distance, 14 kV applied voltage, −2 kV collector, 0.7 mL/h flow rate, 30–40% relative humidity). These baseline parameters were selected based on prior literature and preliminary tests to achieve stable jets with the PEO/alginate system. They were kept constant for all formulations to allow a fair comparison of the solvent effects on spinnability. We note that while these conditions were effective for spinning the ethanol-based extracts, extensive parameter optimization was not the focus of this study. Minor adjustments (such as slightly reducing the flow rate and varying the applied voltage) were explored during trials with NADES-based extracts; however, these did not overcome the fiber formation issues. A more comprehensive optimization of electrospinning parameters may be pursued in future work to improve reproducibility and fiber quality further. 

### 2.9. FTIR Characterization

The FTIR spectra of the samples were obtained using an IRAffinity-1S apparatus (Shimadzu, Kyoto, Japan) in ATR mode, with the range of 4000–400 cm^−1^ and a resolution of 4, with 256 scans for each sample.

### 2.10. Scanning Electron Microscopy

Nanofiber mats of (S)Et5%, (S)Et20%, (S)1CA5%, (S)2CA5% and (S)3CA5% were analysed with a scanning electron microscope (SEM). SEM observations were carried out on a Quanta 250 microscope (FEI, Hillsboro, OR, USA) equipped with a secondary electron detector. Samples were sputter-coated with Au (~20 nm, 99.99% purity). A working distance of approx.10 mm and a vacuum below 10^−4^ Pa were maintained. The nanofiber diameters were manually measured by using the ImageJ 1.54g software (National Institutes of Health, Bethesda, MD, USA) from 100 randomly selected fibers.

### 2.11. Preparation of Alginate-Based Films

The solution of 4% (*w*/*v*) Alg was prepared by dissolving Alg in distilled water at room temperature. Two types of alginate-based films were produced: one with a 5% (*w*/*w*) addition of (E)Et and one with 5% (*w*/*w*) addition of (E)0CA. A mixture of Alg, extracts, and one drop of glycerol was prepared and stirred until completely homogenized. Possible air bubbles were removed using an ultrasonic probe. Then, 12 g of each solution was poured onto the Petri dishes and left to dry for 2 days. After this time, each film was cross-linked with a calcium chloride solution (0.2 M) for 30 s and left to dry completely.

### 2.12. Verification of the Usefulness of Freshness Indicators in Food Testing

Three anthocyanin-based freshness indicator systems under real meat storage conditions (12 days, refrigerated) were compared to determine their color-change behavior, sensitivity, and stability. The systems included: (i) electrospun (S)Et5%(A) and (S)Et20%(B); (ii) cast calcium alginate films with 5% of (E)Et (C); (iii) cast calcium alginate films containing a 5% of (E)0CA (D). The NADES extract was integrated via a film (rather than fibers) because, despite superior anthocyanin yield and stability, its high viscosity and low volatility impeded electrospinning. All indicators were placed with fresh chicken meat and observed over 12 days to record color changes and correlate them with spoilage progression.

## 3. Results

### 3.1. Total Polyphenol and Anthocyanin Content in Red Cabbage Extracts

ChCl-based deep eutectic solvents are considered a safe, efficient, and effective alternative to traditional extraction methods for anthocyanin extraction. Compared to conventional solvents such as ethanol or methanol, ChCl-based deep eutectic solvents, when paired with suitable HBD (including 1,3-butanediol, glycerol, citric acid, lactic acid, and propylene glycol), facilitate higher or equivalent yields of anthocyanin extraction. [15,18,24,25,26]. The acquired TAC and TPC values are presented in Table 2.

The lowest total polyphenol content was recorded in extracts formulated with a 50% ethanol solution and a combination of ChCl:citric acid:water (1:2:6) with ethanol at a ratio of 90:10 (designated as (E)1CA). Deep eutectic solvents were more efficient in extracting polyphenols from red cabbage compared to conventional ethanol. The limited efficacy of pure ethanol substantiates the superior performance of NADES, but the unsatisfactory outcome of the 90% NADES mixture is likely attributable to the solvent’s excessive viscosity. For both NADESs, the highest TPC and TAC values were obtained with 30% ethanol in the solvent mixture. The addition of 30% ethanol likely reduces the viscosity of the solution more effectively than 10%, facilitating the penetration of phenols and anthocyanins from the plant matrix. However, too high a proportion of ethanol worsened the results, with both TPC and TAC decreasing at 50% ethanol ((E)3CA, (E)3LA). This can be explained by the disruption of eutectic properties when NADES is excessively diluted. There are reports that the addition of >50% of a polar diluent (e.g., water) destroys the supramolecular structure of NADES, effectively turning it into a simple solution of its components [27], Our findings demonstrate that the impacts of this phenomenon are most evident for ChCl:lactic acid (1:1), where the (E)3LA (with the incorporation of ethanol 1:1) had a significantly lower total anthocyanin content of 0.047 mg/mL compared to (E)2LA, which recorded 0.205 mg/mL. The correct incorporation of 30% ethanol considerably reduced the viscosity and improved the extraction efficiency of NADES while maintaining its unique characteristics. The (E)2CA demonstrated the most outstanding total phenolic content and total antioxidant capacity values, with TAC recorded at 0.312 mg/mL, about double that of the other samples. This outcome is unexpected, given that NADESs with lactic acid are generally considered more efficient for anthocyanin extraction than those containing citric acid in the literature. The studies demonstrate that the addition of ethanol significantly improved the effectiveness of high-viscosity NADES produced from citric acid, overcoming its early shortcomings.

### 3.2. FTIR Characterization

**Figure 1:**
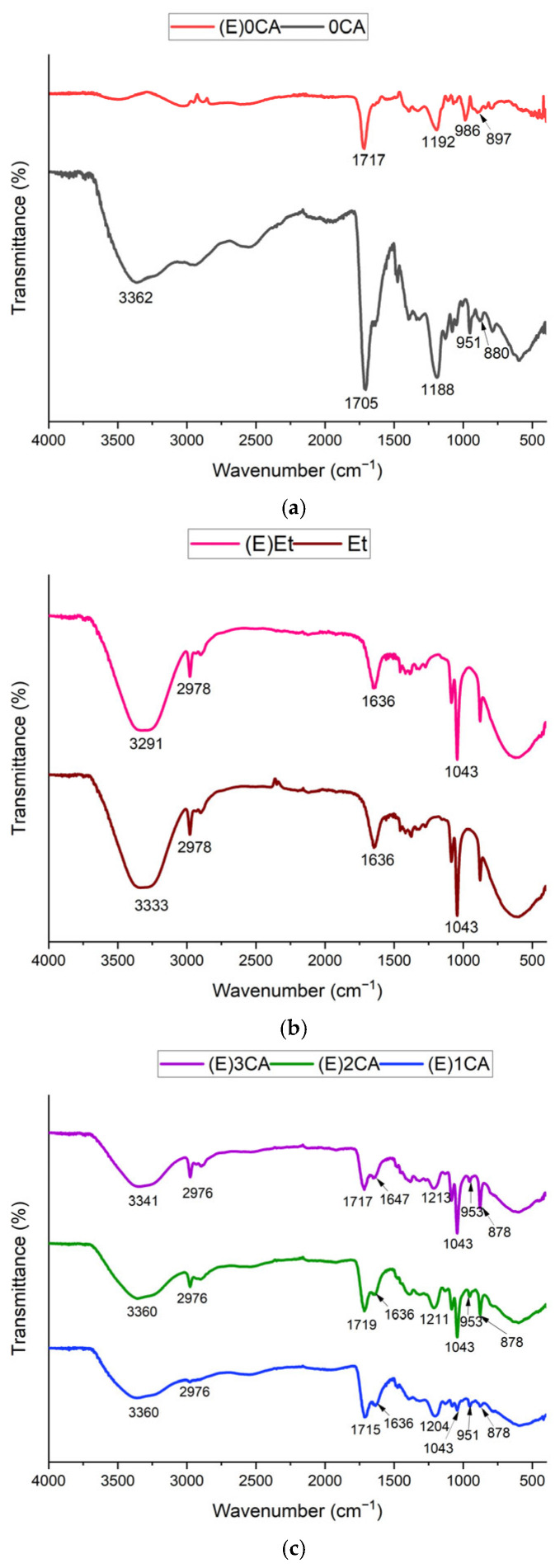
FTIR spectra (Transmittance (%) vs. wavenumber) of: 0CA and (E)0CA (**a**); Et and (E)Et (**b**); and (E)1CA, (E)2CA and (E)3CA (**c**). Spectra in each panel have been offset vertically for clarity, and the *y*-axis is labeled as ‘Transmittance (%)’.

The structural characteristics of NADESs and extracts were investigated with FTIR spectroscopy (Figure 1). FTIR spectra showed the presence of the characteristic functional groups: O-H stretching (3400–3200 cm^−1^), C-H stretching (3000–2800 cm^−1^), C=C allyl stretching vibrations and bending vibration of the H-O-H angle (1660–1610 cm^−1^), C=O stretching of carboxylic acid groups (1700–1750 cm^−1^), stretching bands of C-C and C-O (1300–1000 cm^−1^), C=C allyl wagging vibration, = CH_2_ wagging vibration (1000–900 cm^−1^) and C-C stretching vibrations (878 cm^−1^) [25,28].

For 0CA solvent, the peak indicating O-H stretching at 3362 cm^−1^ suggests the presence of water and hydrogen bonds between ChCl and citric acid. However, after extraction in (E)0CA the spectrum shows a lack of strong OH stretching bands, suggesting that -OH groups are significantly removed (Figure 1a) [29]. Because the red cabbage material became sticky during extraction and the extract was difficult to separate from the residue, it is possible that most of the aqueous fractions remained trapped within the plant residue. The upward shift in the wavenumber of the carboxylic groups from 1705 cm^−1^ in 0CA solvent to 1717 cm^−1^ in (E)0CA extract was observed, as well as the increase in C-O bending from 1188 cm^−1^ to 1192 cm^−1^. This indicates a weakening of the original hydrogen bonding in the 0CA system, connected with the phenomenon called ‘*blue shifting*’ of hydrogen bonds [30].

The spectra of Et and (E)Et are similar to each other (Figure 1b). The only difference appears in the OH stretching peaks, where for Et it is 3333 cm^−1^ and 3291 cm^−1^ for (E)Et. This decrease in wavenumber after extraction indicates strengthening of H-bonding in OH groups [31]. A similar spectrum was obtained for red rice, where authors confirmed the existence of aromatic C=C bonds at 1639 cm^−1^, C-O bonds at 1046 cm^−1^ and C-H stretching at 2928–2977 cm^−1^ and concluded that extracts have anthocyanin compounds with hydroxy groups and aromatic groups [32]. However, the obtained IR spectrum for the solvent (Et) suggests that peaks appear mainly because of the presence of ethanol and water. The peaks at 1636 cm^−1^ are most probably from bending vibrations of water [31].

The spectra obtained for extracts containing both NADESs and ethanol are shown in Figure 1c. According to the rule, maxima located around 3200 cm^−1^ vibrations of strongly H-bonded OH groups, 3450 cm^−1^-weakly H-bonded OH groups and 3650 cm^−1^ mean vibrations of free OH groups without H-bonding [31]. For (E)1CA and (E)2CA, wavenumbers (3360 cm^−1^) are similar to those obtained for solvent-0CA (3362 cm^−1^). Peaks around wavelength of However, for (E)3CA, the maximum is located at 3341 cm^−1^, suggesting the strengthening of the H-bonding with the increase in ethanol content. The increases in wavenumber from 1705 cm^−1^ (for 0CA) to 1715–1719 cm^−1^ for carboxylic groups and from 1188 cm^−1^ (for 0CA) to 1204–1213 cm^−1^ may be caused by weakening of the original hydrogen bonding in 0CA solvent due to blue shifting and creation of possible new interactions between the solvent and active compounds [30]. There is no shifting for groups typical of ethanol, such as C-H stretching at 2976 cm^−1^ and C-O bonds at 1043 cm^−1^. Similar spectra were shown in the literature [25,29]. The differences may be caused by the addition of ethanol and water, and a different molar ratio of ChCl to citric acid.

### 3.3. Electrospinning of PEO/Alginate Polymers with Red Cabbage Extracts

#### 3.3.1. Viscosity of Electrospinning Solutions

**Figure 2:**
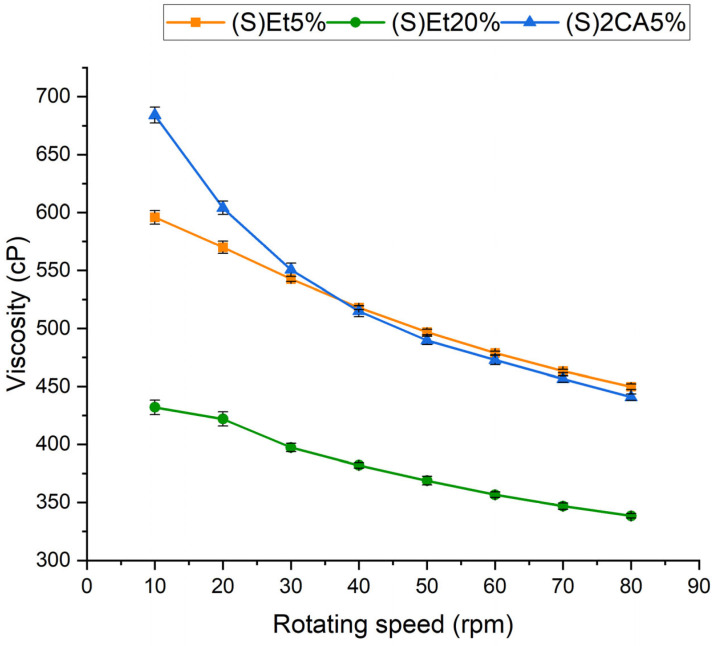
Viscosity profiles of electrospinning solutions.

The electrospinning process requires the appropriate selection of many parameters to enable the successful production of nanofibers. One of the most important is the viscosity of the electrospinning solution. Solutions with too low viscosity prevent proper stretching of the polymer jet, causing electrospraying instead of electrospinning. On the other hand, the viscosity cannot be too high because it can cause clogging of the spinneret [33]. The viscosity of the electrospinning solutions: (S)Et5%, (S)Et20% and (S)2CA5% were evaluated (Figure 2).

Only one electrospinning solution with NADES extract was tested for viscosity, which was caused by droplet formations during electrospinning and problems with fibers production. Therefore, the solution with the highest TAC was chosen. The viscosity of the (S)2CA5% solution decreases much faster with increasing rotating speed for lower rotating speeds (10–40 rpm). At 10 rpm, the viscosity for this solution is the highest (approx. 680 cP) among all tested solutions. For electrospinning solutions, the correlation between viscosity and rotating speed is more linear for those without the addition of NADES extract. Also, higher content of ethanol-based extract in the electrospinning solution results in lower viscosities, which is caused by higher ethanol concentration in the solution. For rotating speed from 30 to 80 rpm, the viscosities of the (S)Et5% and (S)2CA5% solutions are at a similar level (550–450 cP). In all three cases, the viscosity decreases with increasing rotating speed, indicating a shear-thinning character of the solutions, which is typical for solutions containing Alg and PEO [34].

#### 3.3.2. Fibers Morphology 

**Figure 3:**
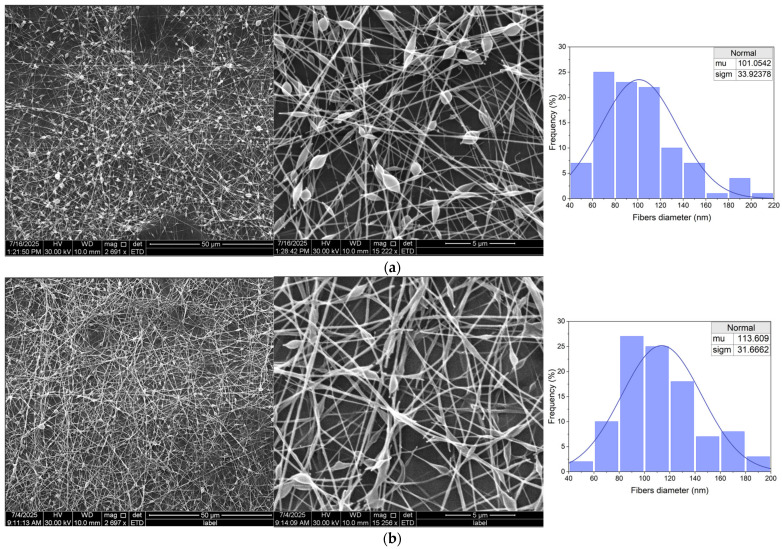
SEM images and diameter distribution of electrospun fibers produced from (S)Et5% (**a**) and (S)Et20% (**b**) solutions (fiber diameters measured from 100 fibers per sample).

**Figure 4:**
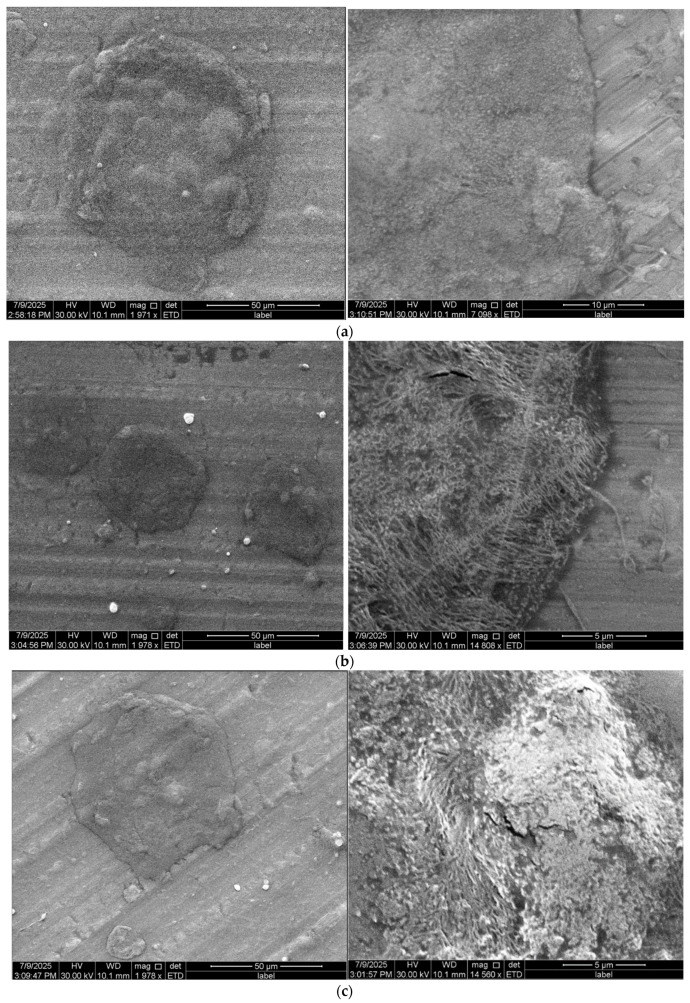
SEM images of electrospun mats produced from (S)1CA5% (**a**), (S)2CA5% (**b**) and (S)3CA5% (**c**) solutions.

Table 3 shows a comparison of fiber morphology observations for different contents of the tested extracts. Electrospinning of anthocyanin extracts from red cabbage, prepared in a 50% ethanol solution, allowed for the production of continuous nanofibers with a structure dependent on the concentration of the extract in the spinning solution. In the case of a 5% extract, SEM revealed the presence of thin, well-stretched, and separated nanofibers (Figure 3a). These fibers were smooth, cylindrical, and had an average diameter of 101 nm (σ = 33.9 nm). The structure of the fibers was very regular, with few small beads. For (S)Et20%, the fibers were also continuous, but noticeably thicker and more morphologically diverse (Figure 3b). The average diameter increased to 113.6 nm (σ = 31.8 nm), and in addition, numerous beads-on-string and local thickening of the fibers were visible. Some of the fibers showed irregularities in shape, flattening, narrowing, and branching, which may indicate lower jet stability and approaching the spinning efficiency limit at this extract level. In both cases, the structure of the mat retained its spatial character, but better morphological parameters (thinner, smoother, and more uniform fibers) were obtained for the sample with 5% extract. The bead-on-string defects and fiber irregularities observed at higher extract loading (e.g., in the 20% ethanol-extract fibers) can be attributed to a reduction in the effective polymer entanglement of the spinning solution. In this case, adding a larger volume of extract (which is mostly solvent) dilutes the polymer, lowering the viscoelastic chain network density and leading to beaded, less uniform fibers. Such defects can be mitigated by increasing polymer concentration or molecular weight to restore sufficient chain overlap, or by optimizing electrospinning parameters (voltage, flow rate, working distance) to stabilize the jet [35]. These strategies ensure the solution reaches the critical entanglement threshold required for uniform fiber formation. SEM micrographs for all five formulations are included in Figure 3 and Figure 4 at two magnifications. Additional images acquired at different resolutions are provided in the Supplementary Materials (Figure S1) to illustrate morphological details further.

Completely different results were obtained for extracts prepared using citric acid-based NADES, even after the addition of ethanol (Figure 4). At the lowest ethanol content (10%), SEM did not show the presence of separate fibers; instead, a fused matrix structure resembling a membrane or aggregates was observed. Thickened and flattened structures were visible. The addition of 30% ethanol only partially improved this effect, with individual fiber-like segments appearing, but these were short, thick, and often connected or stuck together at the point of contact. During electrospinning, the fibers were irregular, and the presence of numerous beads and local breaks indicated the instability of the process (observed under an optical microscope immediately after fiber production). Even with 50% ethanol in the extract, the structure did not achieve a quality comparable to that of ethanol extracts. Moreover, the instability of a jet was observed during the electrospinning of NADES-based extracts, which led to droplet formation. Modifying the electrospinning parameters, such as applied voltage and flow rate, did not improve these effects.

### 3.4. Evaluation of Indicator Performance on Deteriorating Chicken Meat

**Figure 5:**
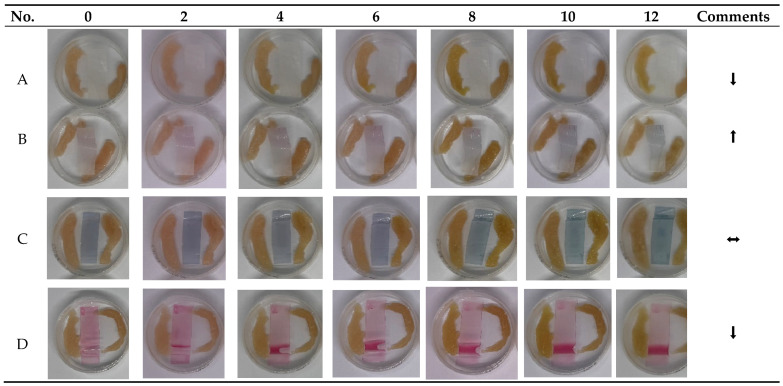
Evaluation of the sensitivity of different materials (**A**–**D**) to chicken meat spoilage over 12 days. (**A**) Electrospun PEO/alginate nanofibers with 5% ethanol-based extract; (**B**) electrospun PEO/alginate nanofibers with 20% ethanol-based extract; (**C**) calcium alginate film with 5% ethanol-based extract; (**D**) calcium alginate film with 5% NADES-based extract (ChCl:citric acid). Arrows indicate the effectiveness of the color change response (⬆ indicates the strongest, fastest response; ⬌ indicates a moderate reaction; ⬇ indicates minimal or no color change).

A 12-day refrigerated meat storage test was conducted to compare the sensitivity, responsiveness to spoilage volatiles, and stability of three anthocyanin-based freshness indicators: electrospun PEO/Alg nanofibers with 5% and 20% ethanol extracts (Figure 5A,B, alginate films with ethanol extract (Figure 5C, and alginate films with NADES extract (ChCl:citric acid, Figure 5D. The electrospun nanofibers (B) exhibited a continuous and progressive color transition from a pale purple hue toward blue-green tones, consistent with the accumulation of basic volatile compounds released during meat spoilage. A visible shift was already noticeable between day 4 and 6 of storage, with intensified blue-green coloration observed by day 10–12. The higher anthocyanin loading (20%) was more effective in generating a clear visual response. In comparison, the lower concentration (5%) resulted in more uniform fibers (sample A) but did not produce a noticeable color change. The nanofibrous structure, characterized by high surface area and porosity, enabled early detection of freshness decline. However, this high sensitivity may require future calibration or protective coating strategies to prevent premature warnings in borderline-fresh products. The alginate film with ethanol extract (C) exhibited a gradual and diffuse color transition during the 12-day storage period. Initially, the film displayed a purple-blue hue, indicating partial deprotonation of anthocyanins in the absence of acid stabilization. Over time, the color slowly shifted toward a dull blue-gray by day 10–12, reflecting moderate sensitivity to spoilage volatiles. The chromatic response, while present, was less vivid and less defined than in the electrospun system, which may limit its practical visibility. The alginate film containing the NADES-based extract (D) showed high initial color intensity, with a vivid red-purple hue attributed to the low pH of the ChCl:citric acid matrix stabilizing the anthocyanins in their flavylium form. However, throughout the entire 12-day storage period, the film exhibited no visible color change, indicating a lack of responsiveness to spoilage volatiles.

## 4. Discussion

The results obtained indicate that natural deep eutectic solvents may outperform traditional organic solvents in the extraction efficiency of phenolic compounds. In our studies, we have shown that pure ethanol extracts polyphenols from red cabbage less efficiently (e.g., TAC only approx. 0.014 mg/mL, Table 2). Similar conclusions can be drawn from the literature, for example, the extraction of anthocyanins from black bean husks using NADES based on ChCl and citric acid resulted in a 26% higher yield of anthocyanins than a conventional 75% ethanol solution [36]. Studies on berries have also shown that NADES acids (e.g., ChCl with citric acid and glycerol) are at least as effective as classic solvents (methanol:water:formic acid) in extracting anthocyanins [25]. Unlike pure ethanol, which usually requires acidification (e.g., HCl) to prevent anthocyanin degradation, DES containing acids (lactic, citric, tartaric, etc.) themselves maintain strongly acidic conditions favorable for the extraction of these compounds. This partly explains the high efficiency of NADES in recovering pigments from red cabbage.

The key factor influencing the efficiency of extraction using NADES is its viscosity. Our results confirm that excessive viscosity limits mass transfer and hinders interactions between solvent molecules and extracted compounds. This phenomenon has been previously observed for many NADES. Due to the extensive network of hydrogen bonds within these solvents, their viscosity can be very high, which reduces the efficiency of phenolic compound extraction. Excessive viscosity hinders the penetration of components and the formation of hydrogen bonds between electron donors and acceptors within the solvent, which limits the extraction efficiency [27]. The addition of a small amount of ethanol (10%) may be insufficient to reduce the viscosity of NADES significantly, hence the TPC for (E)1CA remained low. The literature emphasizes that a strong hydrogen bond network is responsible for the high viscosity of NADES, and reducing this viscosity (e.g., by adding a diluent or heating) increases the efficiency of extraction [37,38]. This confirms the observed improvement in results after adding a larger amount; already, 30% ethanol significantly increased the extraction efficiency. However, there are certain limitations to the dilution of eutectic solvents; too much water can disrupt the structure of NADESs by weakening or breaking hydrogen bonds. Above a specific dilution limit, the eutectic structure is disordered, and the solvent loses its unique properties resulting from the hydrogen bond network, as the molecules of the additional solvent compete for these bonds [39]. Research indicates that at water contents > 50%, the mixture behaves more like a simple solution of its components than a DES [40]. Our observations are consistent with this mechanism; we obtained the best results (TPC and TAC) for a moderate ethanol content (30%), while too high a content (50%) resulted in a decrease in extraction efficiency. In the case of anthocyanin extraction from red cabbage, the optimal solution was therefore a compromise, sufficient ethanol addition to reduce the viscosity of NADES, but not so much as to lose its advantages.

The most surprising result of the study was the high efficacy of the (E)2CA extract, which achieved the highest anthocyanin content (approx. 0.312 mg/mL), surpassing other NADES systems (0.185–0.205 mg/mL) and ethanol (0.014 mg/mL). Although the literature often indicates greater effectiveness of solvents with lactic acid [41,42], our results contradict this trend, showing that appropriately modified NADES with citric acid may be more effective. The addition of ethanol played a key role in reducing viscosity and improving the diffusion of compounds while maintaining an acidic environment. These results highlight the importance of synergy between DES composition and co-solvent for optimizing extraction efficiency.

The FTIR results indicate that extraction led to modification in OH bonding (3400–3200 cm^−1^). The decrease in wave number for both only ethanol extracts and NADES-ethanol extracts compared to the spectra of solvents suggests strengthening of H-bonded OH [31]. This occurred most probably because of the new interactions between solvents and extracted active compounds. The effect of blue shifting that appeared for NADES-based extracts indicates that new interactions replaced the original hydrogen bonds [30].

Analysis of the viscosity of the electrospinning solutions indicates that the viscosity of the solution with NADES-based extract is significantly higher than that of the solutions with only ethanol-based extracts, but only at low rotating speed. Higher addition of ethanol and water-based extract caused a significant decrease in viscosity. Since viscosity is related to the molecular weight of polymers, the increased addition of ethanol or water reduces the concentration of polymer in the electrospinning solution, leading to lower viscosity [43]. FTIR analysis indicates that extraction-induced modifications in O-H bonding are consistent with the formation of new interactions between NADES constituents and the extracted polyphenols. These additional hydrogen-bonding interactions likely reinforce the supramolecular network of the system, leading to a pronounced increase in viscosity of the NADES-containing electrospinning solution. The resulting highly interconnected hydrogen-bonded matrix reduces molecular mobility. It hinders jet elongation, which provides a mechanistic explanation for the inability of these solutions to form continuous nanofibers during electrospinning.

The observed differences in fiber morphology indicate the extent to which the choice of solvent affects the electrospinning process. Our results are consistent with the literature. In a study [20], electrospinning of anthocyanin extracts using NADES based on ChCl and xylitol was described, and the formation of a merging, discontinuous structure was also noted, despite it being referred to as a fiber. The diameters of the obtained structures reached up to 5 µm, corresponding to unusually thick fibers. In some of their experiments, electrospinning resulted in the formation of a film rather than fibers, confirming that the problem is reproducible and relates to the very nature of NADES as a solvent. In our study, the lack of fiber formation is mainly due to the hydrophilicity, hygroscopicity, and low volatility of NADES. Components such as ChCl, organic acids, and glycerol, present in popular NADESs, retain water and evaporate very slowly, which prevents rapid stiffening of the stream during electrospinning. As a result, the fibers remain moist and merge, forming an amorphous matrix. Although we did not directly characterize the PEO/alginate blend in this study, the literature confirms that hydrogen bonding between PEO ether groups and alginate hydroxyl groups facilitates chain entanglement and fiber formation. Caykara et al. demonstrated by FTIR, DSC, TGA, and AFM that such PEO–alginate hydrogen bonding improves miscibility, modifies crystallinity, and enhances the mechanical properties of blend films [44]. Vigani et al. reported that blending alginate with PEO enhances electrospinnability and improves fiber mechanical properties through such interactions [45], while a recent review emphasized polymer blending with PEO as one of the most effective strategies to overcome alginate’s intrinsic limitations in electrospinning [22]. In our system, these intermolecular interactions likely supported the successful spinning of ethanol-based extracts. In contrast, the high ionic strength and water-binding capacity of NADES may have disrupted the PEO/alginate hydrogen-bond network, reducing chain entanglement and resulting in fused, irregular morphologies. Too much water or glycerol in the solution can promote the formation of irregular, melted structures instead of continuous fibers. Although a moderate addition of water can improve fiber orientation, exceeding the optimal level disrupts the spinning process [46]. The addition of glycerol increases the elasticity and plasticity of the fibers, but its excess can cause the formation of larger diameter structures and even lead to fiber discontinuity or the formation of coatings [47]. Extracts obtained using NADESs have high viscosity and conductivity. This helps to maintain a stable jet, but excessive viscosity and a high amount of dissolved substances lead to spinning instability, e.g., through needle clogging, bead formation, or sticking. In our samples, such phenomena were observed in the form of irregular structures, thick fibers stuck together in a uniform mass, with numerous defects. 

Despite the higher content of active compounds (TAC, TPC) in NADES extracts, it was not possible to obtain stable nanofibers from them. On the other hand, ethanol extracts, although containing fewer active ingredients, enabled the formation of homogeneous fibers. This is because the properties determining extraction efficiency (e.g., solubility) differ from those necessary for electrospinning (viscosity, conductivity, polymer entanglement [48,49]. Solutions with high extraction efficiency (e.g., with a large amount of NADES, water, or glycerol) may have physicochemical properties that hinder spinning, leading to the formation of droplets, beads, or “melted structures” instead of continuous fibers. Successful electrospinning requires reaching the so-called critical entanglement concentration of the polymer; below this value, no fibers are formed [35], even if the extract is rich in bioactive compounds. Obtaining nanofibers from NADES extracts requires modification of the spinning strategy or the use of alternative forms, such as films or microcapsules.

Among the evaluated systems, the electrospun nanofibers, specifically sample B containing 20% extract, demonstrated the most significant and sustained color shift from pale purple to blue-green, commencing as early as days 4 to 6. This transition coincided with the accumulation of fundamental spoiling volatiles, including ammonia and amines, often linked to microbial growth surpassing 10^7^ CFU/g [50]. The nanofibrous structure provided a substantial surface area and porosity, improving gas–solid interactions and enabling fast proton exchange with the anthocyanin matrix. The lower-loading version (A) yielded more uniform and stable fibers; nevertheless, the reduced dye concentration resulted in no observable color change, indicating that a minimum threshold is required to elicit a noticeable chromatic alteration. The ethanol-extracted alginate film (C), produced without pH modification, demonstrated a gradual, diffuse color alteration commencing approximately on day 6. Initially purple-blue, the film evolved to a muted blue-gray hue by day 12. The feeble and inconsistent signal was probably due to partial deprotonation of the anthocyanins within the neutral matrix and gradual pigment degradation over time. In the absence of acid stabilization, anthocyanins exhibit increased susceptibility to oxidative and thermal degradation, resulting in color loss and reduced contrast. Conversely, the alginate film infused with the NADES extract (D) exhibited superior pigment retention and vibrant initial coloration attributable to the acidic environment established by citric acid. The robust buffering capacity of the NADES matrix likely mitigated pH changes at the sensor contact, hence limiting structural modification of the anthocyanins. Consequently, no color change was seen during storage, even at advanced levels of deterioration. This result underscores a significant constraint of heavily buffered systems, which, despite their chemical stability, can hinder the sensor’s sensitivity to subtle environmental variations [51].

The results of this study confirm that while NADES-based systems offer superior extraction efficiency and pigment stabilization, their application in freshness sensors requires careful tuning of matrix reactivity. Although the NADES extract exhibited high anthocyanin content and vivid coloration, its strong buffering capacity hindered the indicator’s responsiveness to spoilage volatiles. In contrast, electrospinning allowed for the successful integration of ethanol-extracted anthocyanins into nanofiber mats with excellent structural uniformity and visible, continuous color transitions, even at low extract loading. This highlights the critical role of material architecture in amplifying local environmental changes. Despite lower anthocyanin content, electrospun systems provided the highest sensitivity and practical usability, enabling early and gradual visualization of meat spoilage. The findings underscore the need to align the extraction strategy, pH environment, and carrier structure to design high-performance anthocyanin-based indicators that offer both stability and timely response under real storage conditions.

Future research should investigate hybrid indicator architectures that integrate the high anthocyanin stability of NADES extracts with the environmental responsiveness of electrospun nanofibers. Multilayer or core–shell configurations may facilitate the separation of stabilization and sensing functions within a unified material system. Optimization of electrospinning parameters, such as the incorporation of co-polymers, surfactants, or rheology modifiers, is necessary to achieve stable nanofiber formation from NADES-rich solutions. Furthermore, the integration of smartphone-based image analysis for real-time ΔE quantification may facilitate the practical application of intelligent packaging. Validation across various food matrices, storage conditions, and packaging types, such as vacuum, is essential to confirm the robustness and commercial viability of indicators.

## 5. Conclusions

This study shows that the choice of extraction solvent has a decisive influence on both the efficiency of anthocyanin recovery and the suitability of the extracts for processing into biopolymer-based indicator materials. The choline chloride–citric acid NADESs modified with ethanol provided significantly higher anthocyanin yields (TAC 0.312 mg/mL compared to 0.014 mg/mL for the conventional 50% ethanol extract) and enhanced pigment stability. However, the intrinsic drawbacks of these solvents (their high viscosity and low volatility) negatively affected the electrospinning process, leading to the formation of coalesced, non-fibrous structures. By contrast, the ethanol-based extract supported the successful electrospinning of continuous PEO/alginate nanofibers with uniform morphology and diameters in the range of 100–115 nm. These nanofibers functioned effectively as freshness indicators under real meat storage conditions, showing clear color transitions that correlated with spoilage onset at days 4–6. Alginate films containing ethanol extracts produced a weaker and delayed chromatic response, while NADES-based films, despite their intense initial coloration, they did not exhibit spoilage-related color changes, most likely due to the strong pH buffering capacity of the NADES matrix. Taken together, the results highlight a critical trade-off: while NADESs markedly improve extraction efficiency and pigment stability, their physicochemical properties hinder fiber formation, underscoring the need to carefully balance extraction performance with material compatibility in the electrospinning process. Possible solutions include modifying NADES formulations with more volatile cosolvents, incorporating rheology modifiers or surfactants to reduce viscosity, using post-spinning impregnation of preformed fibers, or applying alternative immobilization strategies such as multilayer films, microencapsulation, or core–shell fiber architectures.

## Figures and Tables

**Table 1:** Analyzed solvents.

Solvent Code	Ethanol Content (wt%)	NADES Content (wt%)	NADES Composition
0CA	0%	100%	ChCl:citric acid:water (1:2:6)
1CA	10%	90%	ChCl:citric acid:water (1:2:6)
2CA	30%	70%	ChCl:citric acid:water (1:2:6)
3CA	50%	50%	ChCl:citric acid:water (1:2:6)
1LA	10%	90%	ChCl:lactic acid (1:1)
2LA	30%	70%	ChCl:lactic acid (1:1)
3LA	50%	50%	ChCl:lactic acid (1:1)
Et	50%	50% (water)	Ethanol:water (1:1, *v*/*v*)

**Table 2:** Summary of TPC and TAC of the tested extracts.

Solvent	TPC [mg GAE/mL]	TAC [mg C-3-G/mL]
(E)1CA	0.765 ± 0.058	0.149 ± 0.015
(E)2CA	1.644 ± 0.172	0.312 ± 0.025
(E)3CA	1.273 ± 0.120	0.186 ± 0.012
(E)1LA	1.336 ± 0.115	0.184 ± 0.018
(E)2LA	1.223 ± 0.146	0.205 ± 0.009
(E)3LA	1.257 ± 0.109	0.047 ± 0.003
(E)Et	0.760 ± 0.051	0.014 ± 0.001

**Table 3:** Fiber morphology observations.

Extract Type and Concentration in the Spinning Solution	Electrospinning Solution Code	Observations	Assessment of Electrospinning
(E)Et 5%	(S)Et5%	beads-on-string, smooth, continuous fibers,	(+++) stable
(E)Et 20%	(S)Et20%	beads-on-string, less smooth fibers, slight entanglement	(++) moderate
(E)1CA 5%	(S)1CA5%	no fibers, amorphous, irregular structure	(−−−) lack of fibers
(E)2CA 5%	(S)2CA5%	no fibers, compact structure, aggregates	(−−−) lack of fibers
(E)3CA 5%	(S)3CA5%	adhesions, irregular mass, aggregates	(−−−) lack of fibers

## Data Availability

The original contributions presented in this study are included in the article/Supplementary Material. Further inquiries can be directed to the corresponding authors.

## Supplementary Materials

The following supporting information can be downloaded at: https://www.mdpi.com/article/10.3390/ma18194502/s1, Figure S1: Additional SEM images of electrospun mats produced from.

## Abbreviations

The following abbreviations are used in this manuscript:

Alg	Sodium alginate
ChCl	Choline chloride
HBA	Hydrogen bond acceptor
HBD	Hydrogen bond donor
NADES	Natural deep eutectic solvent
PEO	Polyethylene oxide
SEM	Scanning electron microscope
TAC	Total anthocyanin content
TPC	Total phenolic content
