# Supplementary material for: Challenges in Nanofiber Formation from NADES-Based Anthocyanin Extracts: A Physicochemical Perspective
Source: Materials (Basel). 2025 Sep 27;18(19):4502. doi: 10.3390/ma18194502 (PMC12526017; doi:10.3390/ma18194502)
Supplement: Supplementary file 1 [file materials-18-04502-s001.zip › materials-3859078-supplementary.pdf]

Challenges in Nanofiber Formation From NADES-Based Anthocyanin Extracts: A Physicochemical Perspective

Paulina Wróbel [1,*], Katarzyna Latacz [2], Jacek Chęcmanowski [3] and Anna Witek-Krowiak [1,*]

[1] Department of Engineering and Technology of Chemical Processes, Faculty of Chemistry, Wroclaw University of Science and Technology, Gdanska 7/9, 50-344 Wroclaw, Poland
[2] Faculty of Chemistry, Wroclaw University of Science and Technology, Wroclaw, Poland; 272043@student.pwr.edu.pl
[3] Department of Advanced Material Technologies, Faculty of Chemistry, Wroclaw University of Science and Technology, Smoluchowskiego 25, 50-370, Wroclaw, Poland; jacek.checmanowski@pwr.edu.pl
* Correspondence: paulina.wrobel@pwr.edu.pl (P.W.); anna.witek@pwr.edu.pl (A.W.-K.)

Supplementary material

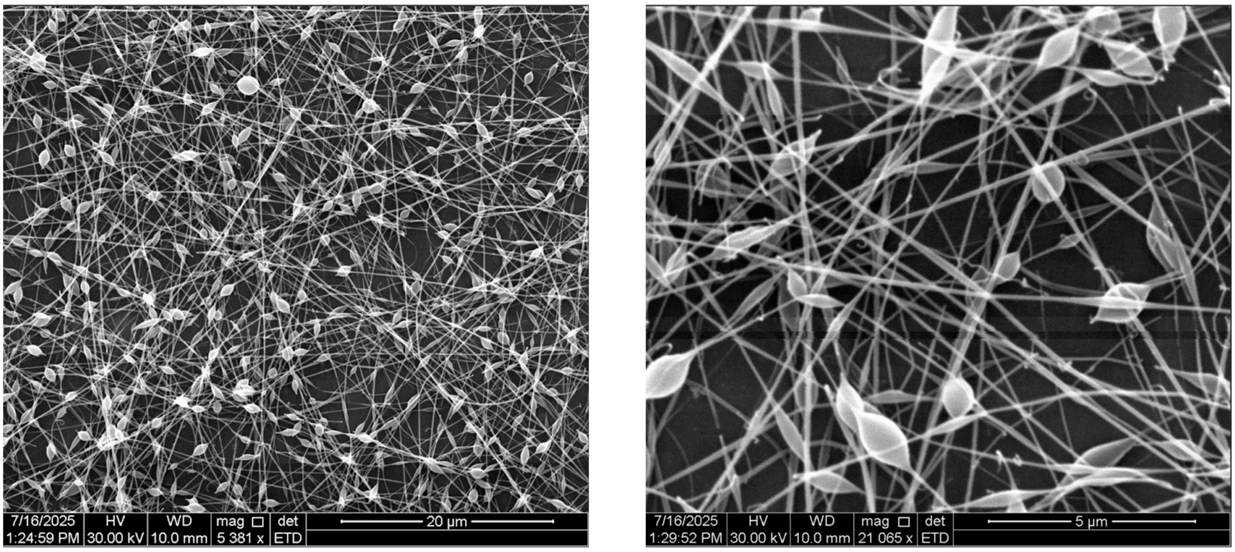

(a)

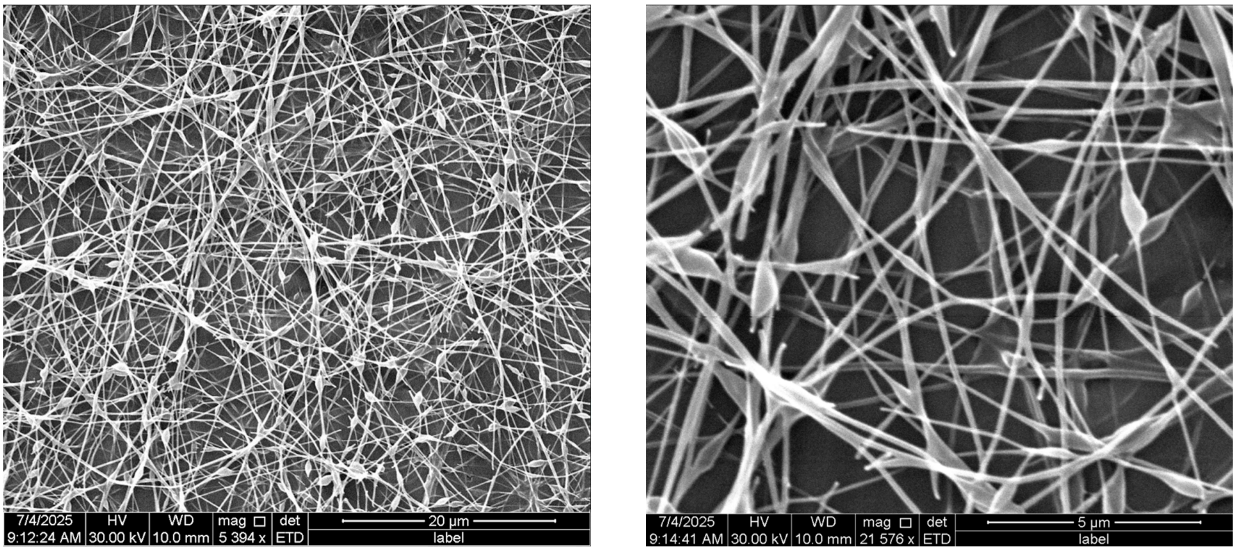

(b)

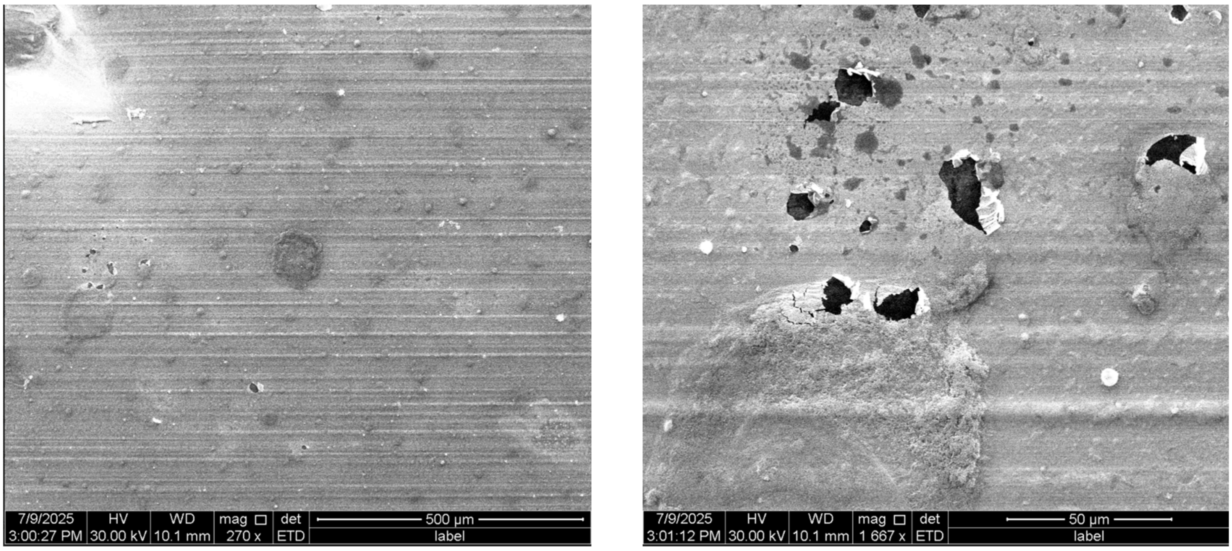
7/9/2025 3:00:27 PM
HV 30.00 kV
WD 10.1 mm
mag ☐ 270 x
det ETD
500 µm
label
7/9/2025 3:01:12 PM
HV 30.00 kV
WD 10.1 mm
mag ☐ 1 667 x
det ETD
50 µm
label

(c)

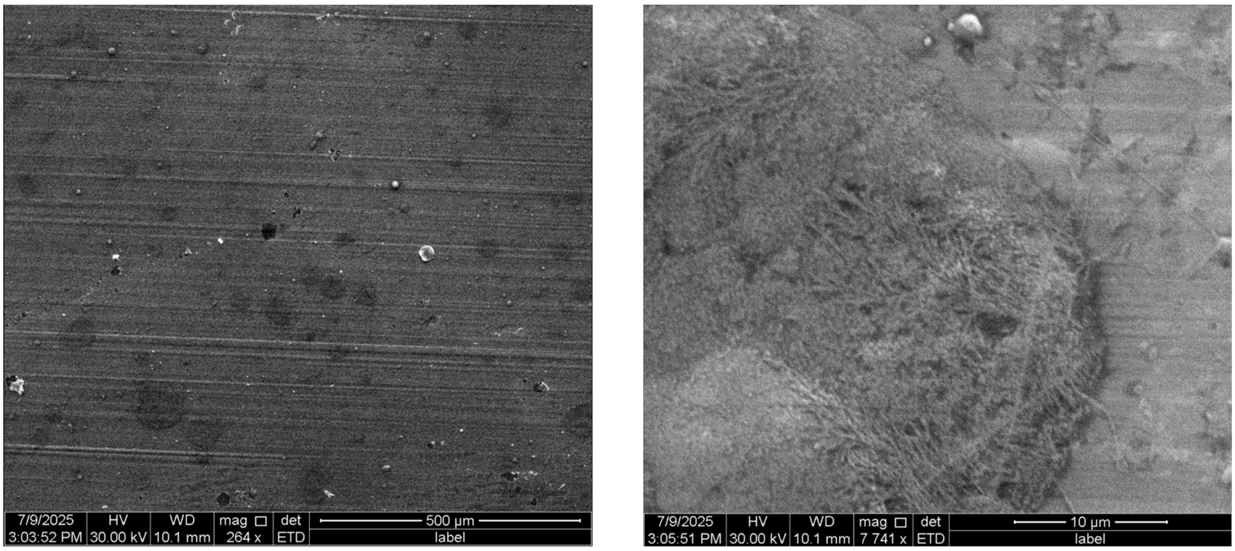
7/9/2025 3:03:52 PM
HV 30.00 kV
WD 10.1 mm
mag ☐ 264 x
det ETD
500 µm
label
7/9/2025 3:05:51 PM
HV 30.00 kV
WD 10.1 mm
mag ☐ 7 741 x
det ETD
10 µm
label

(d)

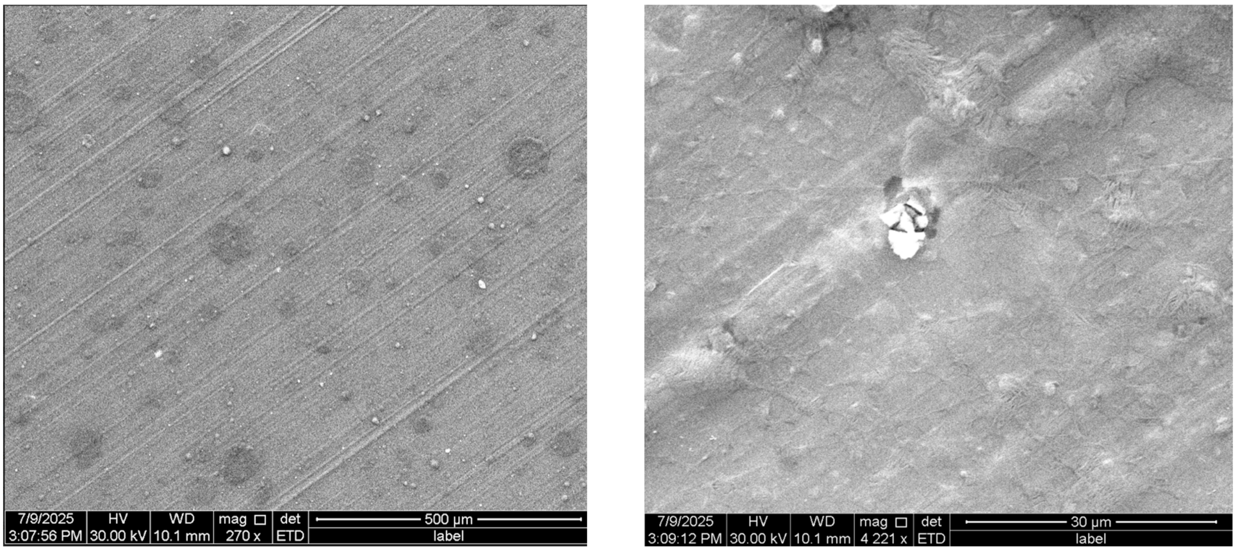
7/9/2025 3:07:56 PM
HV 30.00 kV
WD 10.1 mm
mag ☐ 270 x
det ETD
500 µm
label
7/9/2025 3:09:12 PM
HV 30.00 kV
WD 10.1 mm
mag ☐ 4 221 x
det ETD
30 µm
label

(e)

Figure S1. Additional SEM images of electrospun mats produced from (S)Et5% (**a**), (S)Et20% (**b**), (S)1CA5% (**c**), (S)2CA5% (**d**) and (S)3CA5% (**e**) solutions at different resolutions.
